# Prediction of Sjögren’s disease diagnosis using matched electronic dental-health record data

**DOI:** 10.1186/s12911-024-02448-9

**Published:** 2024-02-09

**Authors:** Jason Mao, Grace Gomez Felix Gomez, Mei Wang, Huiping Xu, Thankam P. Thyvalikakath

**Affiliations:** 1 Department of Biostatistics and Health Data Science, Indiana University Richard M. Fairbanks School of Public Health, 410 W. 10th Street, Indianapolis, IN 46202 USA; 2 Department of Dental Public Health and Dental Informatics, Indiana University School of Dentistry, 1121 W. Michigan Street, Indianapolis, IN 46202 USA; 3 Department of Biostatistics and Health Data Science, Indiana University School of Medicine, 410 W. 10th Street, Indianapolis, IN 46202 USA; 4 Center for Biomedical Informatics, Regenstrief Institute, 1101 West 10th Street, Indianapolis, IN 46202 USA

**Keywords:** Sjögren’s disease, Electronic dental records, Electronic health records, Prediction

## Abstract

**Background:**

Sjögren’s disease (SD) is an autoimmune disease that is difficult to diagnose early due to its wide spectrum of clinical symptoms and overlap with other autoimmune diseases. SD potentially presents through early oral manifestations prior to showing symptoms of clinically significant dry eyes or dry mouth. We examined the feasibility of utilizing a linked electronic dental record (EDR) and electronic health record (EHR) dataset to identify factors that could be used to improve early diagnosis prediction of SD in a matched case-control study population.

**Methods:**

EHR data, including demographics, medical diagnoses, medication history, serological test history, and clinical notes, were retrieved from the Indiana Network for Patient Care database and dental procedure data were retrieved from the Indiana University School of Dentistry EDR. We examined EHR and EDR history in the three years prior to SD diagnosis for SD cases and the corresponding period in matched non-SD controls. Two conditional logistic regression (CLR) models were built using Least Absolute Shrinkage and Selection Operator regression. One used only EHR data and the other used both EHR and EDR data. The ability of these models to predict SD diagnosis was assessed using a concordance index designed for CLR.

**Results:**

We identified a sample population of 129 cases and 371 controls with linked EDR-EHR data. EHR factors associated with an increased risk of SD diagnosis were the usage of lubricating throat drugs with an odds ratio (OR) of 14.97 (2.70-83.06), dry mouth (OR = 6.19, 2.14–17.89), pain in joints (OR = 2.54, 1.34–4.76), tear film insufficiency (OR = 27.04, 5.37–136.), and rheumatoid factor testing (OR = 6.97, 1.94–25.12). The addition of EDR data slightly improved model concordance compared to the EHR only model (0.834 versus 0.811). Surgical dental procedures (OR = 2.33, 1.14–4.78) were found to be associated with an increased risk of SD diagnosis while dental diagnostic procedures (OR = 0.45, 0.20–1.01) were associated with decreased risk.

**Conclusion:**

Utilizing EDR data alongside EHR data has the potential to improve prediction models for SD. This could improve the early diagnosis of SD, which is beneficial to slowing or preventing complications of SD.

**Supplementary Information:**

The online version contains supplementary material available at 10.1186/s12911-024-02448-9.

## Background

Sjögren’s disease (SD) is an autoimmune disease characterized by its effect on exocrine glands, leading to its most common features of dry eyes and dry mouth [1]. It affects an estimated 2 to 4 million Americans, primarily middle aged women, at a 9 to 1 female to male ratio, though this may vary worldwide [2, 3].

SD is a slow progressing disease that currently has no cure, with care focusing on symptom management and prevention of disease complications [4]. Early diagnosis of SD is important as it improves the treatment of and prevents complications related to SD, such as oral complications or lymphoma, a serious complication of SD [5, 6]. Early diagnosis also aids the management of nonspecific symptoms and improves health-related quality of life, which is often low in SD patients [6]. This highlights the critical need for the early diagnosis of SD.

However, early diagnosis of SD is challenging due to the wide spectrum of clinical manifestations, where a quarter of primary SD patients exhibit atypical manifestations [7], nonspecific symptoms such as fatigue and chronic pain [4, 8], and overlapping clinical manifestations with other autoimmune diseases. Prior studies have reported that 50-60% of SD patients have secondary SD, that is, their SD occurs in conjunction with other types of autoimmune diseases such as rheumatoid arthritis and lupus [4, 5]. In addition, there is currently no universally accepted gold standard for the diagnosis of SD. Classification criteria focused on objective tests of SD signs and symptoms have been created to aid clinical research [9, 10] but are less suited for diagnosing atypical or preclinical SD patients compared to diagnosis criteria, which are broad and emphasize accurate diagnosis [11, 12]. Tests based on these criteria may also require seeing multiple medical specialists, potentially delaying diagnosis. These tests do not consider novel antibodies [13] or salivary gland ultrasonography [14], among other tests that may improve the early diagnosis of SD [9, 15]. For these reasons, SD is often diagnosed late [5, 9, 16]. Estimates of the diagnosis delay range from 2 to 12 years between the onset of symptoms and the diagnosis of SD [15, 17, 18].

SD patients experience salivary gland swelling, dental caries, tooth loss, and changes in their saliva as a consequence of decreased saliva flow [19]. Changes in saliva composition due to SD weaken the antimicrobial function of saliva, contributing to caries development, oral infections, and difficulty maintaining good oral hygiene [19, 20]. Despite more frequent dental visits than healthy individuals [21], even with good oral hygiene, SD patients experience more oral issues than healthy patients [19, 22]. These issues may occur in SD patients before clinically significant dry mouth is detected [23], suggesting that oral manifestations may be useful in the early diagnosis of SD.

Electronic health record (EHR) systems are increasingly used for clinical research, particularly in the area of machine learning (ML) [24, 25], taking advantage of the collection of real-world clinical data [26]. Electronic dental record (EDR) data offer much of the same benefits as EHR data but are less often utilized for clinical research [27]. While these systems have historically been siloed from one another, integration of these systems can improve patient care and support clinical research [27, 28].

Few studies have attempted to create prediction models for SD, though ML has been successfully applied to identify other autoimmune diseases that share diagnostic challenges with SD [29, 30]. Using EHR and medical claims data, Dros et al. (2022) classified primary SD with ML based models including logistic regression and random forest [31]. Despite the good discrimination of the models, this study did not separately use patient data prior to the SD diagnosis to predict SD, which is critical for it to be useful in early diagnosis. In addition, there have been no studies that use information from both EHR and EDR to identify SD, despite the potential value of dental information for the early diagnosis of SD.

In this study, we used a highly curated, well-characterized set of integrated EDR-EHR data that describes SD patients to create prediction models for the risk of SD diagnosis. This study is exploratory with the goal of identifying factors significantly associated with the later diagnosis of SD. In focusing on signs and symptoms that appear prior to diagnosis, we hope to identify important variables that can be used to improve the early diagnosis of SD. In addition, we examined whether the inclusion of dental data increases the utility of SD prediction models compared to modeling with medical data alone.

## Methods

### Data source

This study drew EHR data from the Indiana Network for Patient Care (INPC), an EHR data repository that collects and integrates healthcare data from across the state of Indiana [32]. The INPC is one of the oldest and largest health information exchanges in the United States, with over 10 billion clinical observations and 18 million patients, interacting with approximately two-thirds of the residents of Indiana [33]. The EDR data were extracted from the Indiana University School of Dentistry (IUSD) system (axiUm®) and linked to the EHR data using the Regenstrief Global Linkage Algorithm, a deterministic patient matching algorithm [34].

### Study population

**Fig. 1:**
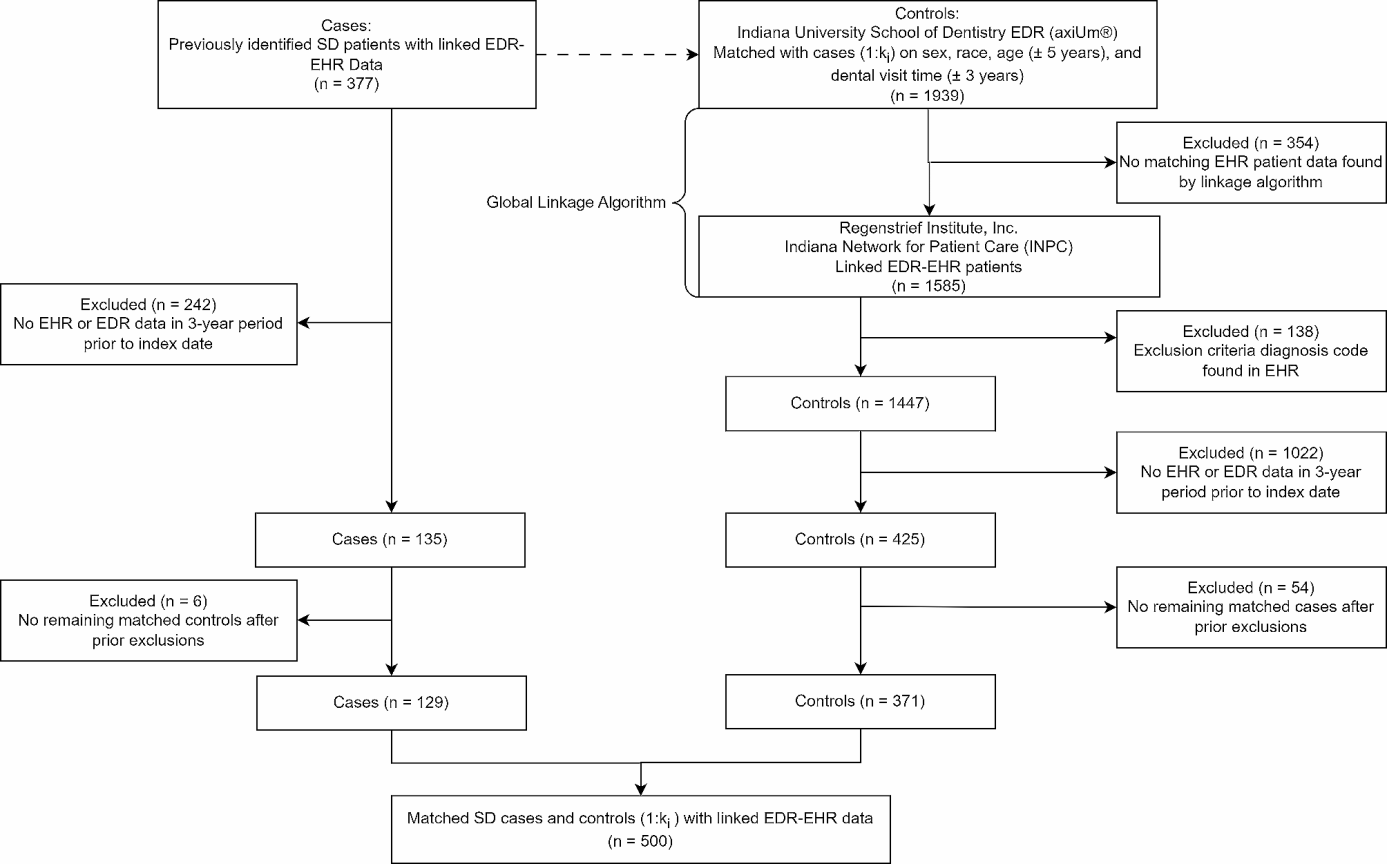
Flow diagram of process to generate study sample. EHR: electronic health record; EDR: electronic dental record; Maximum ki = 6

This retrospective case-control study examines clinical and dental risk factors that are predictive of SD diagnosis. Data were collected for patients who were at least 18 years of age and had records of at least one dental procedure code between June 2005 and January 2021. Chart review was performed for patients with a recorded International Classification of Diseases, Ninth and Tenth Revision (ICD-9/ICD-10) code of SD within their EHR data (ICD-9: 710.2; ICD-10: M35.0 to M35.09) or Regenstrief internal/local concept code 8232 [35]. We considered patients with an SD code in their history as SD cases, confirmed by clinical diagnosis. Patients who did not have a recorded diagnosis of SD were considered controls. Patients with any of the following conditions were excluded from the study: history of head and neck radiation therapy, active hepatitis C infection, acquired immunodeficiency syndrome (AIDS), sarcoidosis, pre-existing lymphoma, amyloidosis, graft versus host disease, and IgG4-related disease. Up to six controls were matched to each case based on sex, race, age at the time of the dental visit (± 5 years), and the dental visit time (± 3 years). For cases, index dates were defined as the date of the first recorded SD code. Controls were assigned the index date of their matched case patient. We examined EHR and EDR data collected in the three-year period prior to the index date so that we could analyze the potential for modeling SD diagnosis risk using predictors found prior to formal diagnosis. Patients with no observed EHR or EDR records in this period were therefore excluded from this study. Cases and controls left without corresponding matches after applying the exclusion criteria were further excluded. Our final study population is matched under 1:k_i_ case to control matching, with up to six controls matched per case. Figure 1 provides an overview of the study sample generation process. We calculated the Cohen’s d statistic based on our sample size to evaluate the effect size our data is powered to detect using SAS PROC POWER.

### Study variables

Variables collected for this study include demographics, comorbidities based on ICD-9/ICD-10 codes, medication history, serological test (antinuclear antibody, rheumatoid factor, anti-SSA, and anti-SSB) history, dental procedures based on Current Dental Terminology (CDT) codes, and symptoms of dry eyes and dry mouth extracted from clinical notes. Based on literature review and expert clinician feedback, we selected potential clinically relevant ICD codes (Additional file 1) and grouped them into categories of comorbidities associated with SD. Comorbidities that were present in less than 3% of patients were excluded from consideration. Drugs commonly prescribed to SD patients were summarized under Medi-Span Generic Product Identifier (GPI) classes to identify drug classes as candidate predictors. For dental procedures, we grouped CDT codes by category of service, categorizing procedures as endodontics, fixed prosthetics, orthodontics, periodontics, preventative, removable prosthetics, restorative, diagnostic or surgical (Additional file 2). Regenstrief Institute’s nDepth™ natural language processing (NLP) tool was used to extract information from clinical notes contained in the HER for the presence of dry eyes and dry mouth using the key terms of dry eye, dry mouth, xerostomia, and hyposalivation. The ConText algorithm [36] was employed to identify and exclude clinical notes in which the key terms were described in a negating or hypothetical context. All nondemographic data were summarized as binary variables, representing any patient level presence in the EHR or EDR data in the three-year period prior to the index date.

### Statistical analysis

Demographic and key analysis variables are summarized with descriptive statistics, presenting frequencies and percentages for categorical variables and means and standard deviations for continuous variables. Due to the matched case-control study design, conditional logistic regression (CLR) was used to evaluate the association between predictors and SD.

We used Least Absolute Shrinkage and Selection Operator (LASSO) regression to select the important variables for the prediction of SD in our models. LASSO is a penalized regression method commonly used in prediction models. LASSO shrinks the regression coefficients of variables that are not useful for prediction to zero, performing model selection and creating models that reduce overfitting while maintaining interpretability [37]. Analyses were performed with the R package *clogitL1*, which fits regularized, LASSO CLR models [38]. We built two models, one using only EHR data and the other using variables from both the EHR and EDR. The performance of our models was evaluated using the concordance index (C-index) proposed by Brentall et al. [39] for the discriminatory ability between cases and controls. We used the C-index rather than the Receiver Operating Characteristic (ROC) curve since CLR cannot be used to generate predicted probabilities, which are needed in the evaluation of sensitivity and specificity and consequently ROC.

We utilized the Cochran-Mantel-Haenszel (CMH) test, which tests the association between two binary variables while controlling for a third [40], to investigate potential confounding relationships among predictors. One variable was excluded due to evidence of significant confounding.

Data analyses were conducted with R version 4.1.0.

## Results

A total of 377 SD cases were identified after chart review [35]. We identified 1939 controls from the IUSD EDR, of which 1585 were successfully linked to patients in the INPC database. We excluded 138 controls due to the presence of exclusion criteria codes. After excluding patients without EHR and EDR data in the three-year period prior to the index date, our final dataset included 129 cases and 371 controls (Fig. 1). Based on a power of 0.8, the Cohen’s d of our sample was 0.287, a small effect size. Table 1 presents patient demographic characteristics as well as EHR and EDR variables included in our multivariable models (Additional file 3). The average age (mean ± SD) of patients in this study was 56.0 ± 16.3, where 82.4% of the sample was 40 years or older at the time of index date. Our sample was primarily female (93.3%) and Caucasian (77.2%). Due to matching, demographic characteristics were balanced between cases and controls with no significant differences between the two groups.

**Table 1:** Descriptive summary of study patient demographics and clinical characteristics by Sjögren’s disease status

Variable	Non-SD Control (*n* = 371)	SD Case (*n* = 129)	Total (*N* = 500)	p-value
Age, mean (std. dev.)	56.2 (16.5)	55.3 (15.8)	56.0 (16.3)	0.231
**Gender, n (%)**				0.996
Female	346 (93.3)	118 (91.5)	464 (92.8)	
Male	23 (6.2)	11 (8.5)	34 (6.8)	
Unknown	2 (0.5)	0 (0)	2 (0.4)	
**Race, n (%)**				0.794
Asian/Pacific Islander	3 (0.8)	2 (1.6)	5 (1.0)	
Black or African American	70 (18.9)	27 (20.9)	97 (19.4)	
Multiracial	3 (0.8)	1 (0.8)	4 (0.8)	
Other/Unknown	6 (1.6)	1 (0.8)	7 (1.4)	
White	288 (77.6)	98 (76.0)	386 (77.2)	
American Indian or Alaska Native	1 (0.3)	0 (0)	1 (0.2)	
**Drug Class, n (%)**				
Antimalarials	4 (1.1)	18 (14.0)	22 (4.4)	7.00E-06
Glucocorticosteroids	59 (15.9)	47 (36.4)	106 (21.2)	1.52E-07
Nonsteroidal Anti-inflammatory Agents (NSAIDs)	88 (23.7)	49 (38.0)	137 (27.4)	0.001
Throat Products - Misc.	3 (0.8)	14 (10.9)	17 (3.4)	2.49E-05
**Diagnosis Category, n (%)**				
Depressive disorder	56 (15.1)	42 (32.6)	98 (19.6)	1.77E-05
Diabetes	60 (16.2)	21 (16.3)	81 (16.2)	0.943
Myalgia and myositis/Fibromyalgia	24 (6.5)	36 (27.9)	60 (12.0)	4.13E-08
Pain in joints	73 (19.7)	58 (45.0)	131 (26.2)	3.10E-07
Systemic lupus erythematosus	5 (1.3)	21 (16.3)	26 (5.2)	1.35E-06
Tear Film Insufficiency	5 (1.3)	21 (16.3)	26 (5.2)	3.66E-06
**Dental Procedure Category, n (%)**				
Diagnostic	323 (87.1)	107 (82.9)	430 (86.0)	0.239
Surgery	68 (18.3)	45 (34.9)	113 (22.6)	8.40E-05
Laboratory Test: Rheumatoid Factor, n (%)	9 (2.4)	18 (14.0)	27 (5.4)	1.18E-05
Dry Mouth, n (%)	10 (2.7)	38 (29.5)	48 (9.6)	1.70E-10
Dry Eyes, n (%)	0 (0)	12 (9.3)	12 (2.4)	1.99e-08

Std. dev.: Standard deviation; p-values from univariable conditional logistic regression models

Diagnostic dental procedures were the most common predictor of interest, present in 86.0% of patients, but did not significantly differ between cases and controls in univariable analysis (*p* = 0.24). Cases and controls differed significantly in univariable analyses of medication history, diagnoses, surgical dental procedures, rheumatoid factor (RF) testing, and presence of dry mouth, with these predictors appearing more frequently in cases. Of note, the presence of dry mouth differed significantly (*p* = 1.7e-10), with approximately 30% of cases reporting dry mouth prior to SD diagnosis compared to only 2.7% of controls.

While building the multivariable predictive model for SD, we found that diabetes was borderline significant with a negative association (*p* = 0.05), even though it was not significant in univariable comparisons (*p* = 0.94). The negative association between diabetes and SD in the multivariable analysis was inconsistent with prior studies that linked metabolic abnormalities with SD [41, 42]. Such a finding is likely a result of overadjustment since diabetes was significantly associated with dry mouth, joint pain, and depressive disorder (*p* = 0.04, *p* < 0.001, and *p* = 0.001 respectively), which are known risk factors for SD. Therefore, we removed diabetes from consideration in our final models.

The results of the multivariable models are shown in Table 2. The multivariable model based on EHR data (model 1) and the model based on integrated EDR-EHR data (model 2) largely overlap in terms of the variables selected. The prescription of lubricating throat product class drugs, joint pain, tear film insufficiency, rheumatoid factor testing, and the presence of dry mouth were significantly associated with an increased risk of SD diagnosis in both models. Based on model 2, the odds for developing SD were 14.97 (95% CI = 2.70-83.06) times higher in patients who were prescribed throat product drugs than in those who were not, 2.54 (95% CI = 1.34–4.76) times higher in patients who experienced pain in joints, 27.04 (95% CI = 5.37-136.26) times higher in patients who experienced tear film insufficiency, 6.97 (95% CI = 1.94–25.12) times higher in patients who were tested for rheumatoid factor, and 6.19 (95% CI = 2.14–17.89) times higher in patients who reported dry mouth.

**Table 2:** Odds ratios of risk of Sjögren’s disease diagnosis according to conditional logistic regression models

Predictor	Odds Ratio (95% Confidence Interval)	p-value
**Conditional Logistic Regression Model Based on EHR Data**		
Drug Class: Antimalarials	1.88 (0.18, 19.51)	0.598
Drug Class: Glucocorticosteroids	1.36 (0.62, 2.97)	0.448
Drug Class: Nonsteroidal Anti-inflammatory Agents (NSAIDs)	1.23 (0.62, 2.45)	0.555
Drug Class: Throat Products - Misc.	13.14 (2.49, 69.26)	0.002
Diagnosis: Depressive disorder	1.29 (0.62, 2.66)	0.494
Diagnosis: Myalgia and myositis/Fibromyalgia	1.79 (0.71, 4.53)	0.215
Diagnosis: Pain in joints	2.36 (1.27, 4.37)	0.006
Diagnosis: Systemic lupus erythematosus	2.60 (0.30, 22.27)	0.382
Diagnosis: Tear Film Insufficiency	32.35 (6.21, 168.63)	< 0.001
Laboratory Test: Rheumatoid Factor	7.09 (2.02, 24.81)	0.002
Dry Mouth	6.91 (2.41, 19.82)	< 0.001
**Conditional Logistic Regression Model Based on Integrated EHR and EDR Data**		
Drug Class: Antimalarials	2.10 (0.18, 24.03)	0.552
Drug Class: Glucocorticosteroids	1.66 (0.74, 3.75)	0.223
Drug Class: Nonsteroidal Anti-inflammatory Agents (NSAIDs)	1.15 (0.57, 2.34)	0.699
Drug Class: Throat Products - Misc.	14.97 (2.70, 83.06)	0.002
Diagnosis: Myalgia and myositis/Fibromyalgia	1.50 (0.58, 3.90)	0.403
Diagnosis: Pain in joints	2.53 (1.34, 4.76)	0.004
Diagnosis: Systemic lupus erythematosus	2.17 (0.23, 20.15)	0.496
Diagnosis: Tear Film Insufficiency	27.04 (5.37, 136.26)	< 0.001
Dental Procedure: Diagnostic	0.45 (0.20, 1.01)	0.053
Dental Procedure: Surgery	2.33 (1.14, 4.78)	0.021
Laboratory Test: Rheumatoid Factor	6.97 (1.94, 25.12)	0.003
Dry Mouth	6.19 (2.14, 17.89)	< 0.001

Model selection was performed using LASSO for conditional logistic regression models

Model 1 provides similar estimates of these effects. Depressive disorder was selected in model 1 with a nonsignificant association with SD, with an odds ratio (OR) of 1.29 (95% CI = 0.62–2.45) when controlling for the other predictors, but it was not selected in model 2. Among variables extracted from the EDR, diagnostic and surgical dental procedures were selected in model 2. Diagnostic procedures were associated with an OR of 0.45 (95% CI = 0.20–1.01) but did not reach statistical significance (*p* = 0.053). Surgical dental procedures were associated with 2.33 (95% CI = 1.14–4.78) times higher odds when controlling for other predictors. Both models show a strong ability to discriminate between cases and controls. Model 1 had a C-index of 0.811 (95% CI = 0.750–0.872) while model 2 had a C-index of 0.834 (95% CI = 0.775–0.893).

## Discussion

To the best of our knowledge, this study is the first to link EDR and EHR data to create a predictive model for the SD diagnosis. Both of our models had a high ability to discriminate between SD cases and controls, with results indicating that the addition of EDR data improves the predictive models that only utilize EHR data. Although the increase in model discrimination was relatively small (2% in C-index), this suggests that the integration of medical and dental health records has the potential to improve the ability to diagnose SD early.

Our models identified several predictors strongly associated with SD, including dry mouth and the presence of other autoimmune diseases, which is consistent with past findings. Evidence of dry eyes and dry mouth, present in approximately 80% of all SD patients [9], was identified via NLP in the EHR clinical notes of approximately one third of our SD cases (Table 2). However, dry eye was not included in our final models, due to its low prevalence in our sample (2.4%). Evidence of dry eyes and mouth may also be captured indirectly within other predictors. For example, fewer SD patients had clinical notes mentioning dry eyes (*n* = 12) than patients who were diagnosed with tear film insufficiency (*n* = 21), another significant predictor, with only four SD patients reporting both. This suggests that there is room for improvement in documenting and identifying dry eyes and dry mouth in clinical notes. In our group’s previous study, 117 of 377 (31.0%) SD cases had no evidence of dry eyes or dry mouth, positing that physician-diagnosed SD can be established without sicca symptoms and tests [35]. This is consistent with findings that oral and ocular objective tests are infrequently performed in clinical practice [43]. Despite this, we found that sicca symptom information derived from an NLP analysis of clinical notes was highly predictive of SD diagnosis. This suggests that unstructured data could be used to identify early occurrences of SD symptoms, aiding the early diagnosis of SD. While we only examined clinical notes within the EHR, this represents a potential avenue toward increasing the utilization of EDR data, in which storing information as structured diagnosis data is less common than in EHRs [44–47].

Antimalarial, glucocorticosteroid, and nonsteroidal anti-inflammatory agent (NSAID) drug prescriptions, as well as pain in joints, are predictors identified by our model that are associated with increased SD diagnosis risk, with the latter three present in 36% or more of cases compared to 15-24% of controls. This indicates that our case patients are experiencing and treating inflammation and pain, symptoms common to autoimmune diseases [48], more frequently than controls. This effect may be mediated by the presence of other autoimmune diseases. In our models, myalgia, myositis, fibromyalgia, and systemic lupus erythematosus were also predictors of SD diagnosis, suggesting that the presence of other autoimmune diseases can be used in SD diagnosis prediction. Nevertheless, overlaps in the clinical and immunological spectrum of SD with other systemic autoimmune diseases make distinguishing between primary SD, secondary SD, and other autoimmune diseases difficult [49]. Serological testing could aid in differentiating the conditions. However, because tests such as rheumatoid factor testing are not unique to SD, care must be taken so that SD prediction models utilizing laboratory data are not predicting a general class of autoimmune diseases. Secondary SD has been studied less extensively than primary SD. Evidence suggests that the clinical phenotypes of an associated autoimmune disease may be affected by the presence of SD. The converse is also true, where patients with secondary SD may express clinical characteristics that differ between associated autoimmune diseases [50, 51]. An improved understanding of secondary SD could also provide information on predictive factors unique to its subclassifications, aiding SD diagnosis.

Our EDR-EHR linked data model found that diagnostic dental procedures may be negatively associated with SD diagnosis. SD patients seek dental care more frequently than healthy patients and require more oral treatments [21], which is consistent with our finding that dental surgical procedures are significantly associated with increased risk of SD diagnosis. The inverse is true for diagnostic procedures, as our SD patients were less likely to have diagnostic procedures than our controls. Patients with more severe oral health problems may be more likely to seek care at the IUSD for known issues and avoid diagnostic procedures they deem unnecessary. This may be because they were diagnosed prior to the three-year period we examined or because they are seeking routine care, including diagnostic procedures, outside of the IUSD, of which we would have no record. Patients with long-standing oral health issues may be more likely to have received diagnoses outside of the examined time period, while healthier patients may be more likely to seek routine diagnostic or preventative dental procedures. As severe oral health issues typically require more expensive treatments, financial considerations may also affect the care-seeking behavior of the patients in our sample, as many patients self-pay for dental treatments at the IUSD, which offers reduced cost dental care to its patients.

Uncommon diseases, such as SD, present additional challenges in utilizing EHR/EDR data to create prediction models. EHR/EDR systems are primarily intended to support patient care and documentation for administrative and patient care purposes, while reusing data from these systems for research is a secondary use case [52, 53]. Because of this, there are significant data quality concerns regarding the secondary use of this data for research, including factors such as the completeness, correctness, and consistency of the data [26, 54]. These concerns are further compounded in the analysis of uncommon diseases. It may be difficult to gather the minimum number of patients needed to construct a robust prediction model for uncommon diseases. In addition, structured data for EHR/EDR is often sparse, containing many codes that are rarely present for individual patients, especially with respect to uncommon diseases. For example, though it was a significant predictor, only 2.4% of our sample reported dry eyes. However, the absence of documentation of a feature does not necessarily indicate the feature itself is absent. In addition, a significant amount of clinical findings are captured in clinical notes rather than structured data [35, 55]. This data is difficult to retrieve without additional processing of clinical notes [46, 47, 54], leaving potentially useful information such as tooth decay data unused. The lack of a diagnostic gold standard, disease heterogeneity, and potential for misclassification also makes accurately capturing SD and other uncommon diseases in the EHR/EDR difficult [56]. Inconsistencies in information may arise from differences in documentation and coding standards between different healthcare settings [54] or while integrating EHR and EDR data [28]. Fitting prediction models to EHR/EDR data is also subject to systematic biases such as selection bias [57, 58].

Another limitation of our study is selection bias due to the criteria we applied to select our sample. Subjects in our study were required to have data from both the INPC EHR and IUSD EDR, with risk factors identified based on the three-year period prior to index date of diagnosis We also required patients to have a minimum amount of analyzable data to be included in our study sample. The IUSD EDR system started collecting data in July 2005, but approximately 16% of our initial 377 cases were diagnosed with SD prior to that date, rendering them ineligible for inclusion in our study sample population. Thus, we have selection bias in our sample arising from missing data, whether it is structural or missing at random [59]. Each of these factors limits the generalizability of our findings outside of our sample population. Techniques such as inverse-probability weighting or multiple imputation could be used to address selection bias caused by missing data, while propensity score matching could be used to reduce the bias introduced by our rule-based case-control matching design [60].

Because of our sample selection procedure, and because SD is uncommon, our study was faced with the limitations due to small sample size. When developing prediction models, sample sizes should be chosen to produce accurate predictions while minimizing overfitting. Larger sample sizes increase the likelihood of building a robust model that can be externally validated, though machine learning models may require even larger sample sizes than traditional models [61]. The wide confidence intervals observed in some of the odds ratios are also a consequence of the small sample size, indicating the precision of such estimates is low and should be interpreted with caution. This is seen in imbalanced predictors such as tear film insufficiency, which was present in approximately 1% of controls compared to 16% of cases. While the association with SD diagnosis may be significant, the effect itself may be overestimated. Future studies should increase the sample size to improve the precision of the estimated confidence intervals or consider techniques designed for imbalanced predictors.

Our study was also limited by its matched case-control design. This design and our small sample size limited our choices for modeling methods. The matched case-control design precluded the possibility of evaluating the effect of matching variables on SD diagnosis risk, though age and gender are known to be significantly associated with SD [62]. We cannot calculate predicted probabilities, which are required to calculate the measures of diagnostic accuracy that are needed to evaluate the calibration of predictive models. Future studies utilizing a prospective study design with a larger sample size are needed to evaluate the risk factors identified in our model for their usefulness in early SD diagnosis. Future work should aim to utilize a large sample of patients to reduce potential biases in the analyses. Exploring study designs beyond the retrospective matched case-control design, such as a prospective cohort study, could be beneficial towards reducing bias and confounding while increasing sample size. While this study largely focused on structured data, further exploration of unstructured data such as caries information, found in EDR clinical notes, could boost a model’s ability to predict SD. Utilizing more advanced modeling methods could improve predictive accuracy and create models with more than one outcome, such as models that delineate primary and secondary SD.

## Conclusions

This study built a prediction model using linked EDR-EHR. It showed, using EDR data from a real-world community-based dental practice, that integrating EHR and EDR data has the potential to improve predictions for SD diagnosis. Future studies are needed to evaluate the generalizability of this approach. This could shorten the diagnosis delay for SD.

### Electronic supplementary material

Below is the link to the electronic supplementary material.

Additional file 1: ICD codes by diagnosis category. A list of ICD codes we identified to be relevant to SD patients, summarized into groups to create analysis variables.

Additional file 2: CDT codes by procedure category. A list of CDT codes and procedure descriptions, grouped by category of service at IUSD, summarized to create analysis variables.

Additional file 3: Variables not selected for inclusion in the final models. A descriptive summary of demographic, drug, diagnosis, dental procedure, serological test history, and clinical note data we used in our study that were not selected into our final models.

## Data Availability

The data that support the findings of this study are available from the Regenstrief Institute, but restrictions apply to the availability of these data, which were used under license for the current study, and so are not publicly available. Data are however available from the authors upon reasonable request and with permission of the Regenstrief Institute.

## Abbreviations

AIDS: Acquired Immune Deficiency Syndrome
CDT: Current Dental Terminology
C-index: Concordance Index
CLR: Conditional Logistic Regression
CMH: Cochran-Mantel-Haenszel
EDR: Electronic Dental Record
EHR: Electronic Health Record
GPI: Generic Product Identifier
ICD-9: International Classification of Diseases, Ninth Revision
ICD-10: International Classification of Diseases, Tenth Revision
INPC: Indiana Network for Patient Care
IRB: Indiana University Institutional Review Board
IUSD: Indiana University School of Dentistry
LASSO: Least Absolute Shrinkage and Selection Operator
ML: Machine Learning
NLP: Natural Language Processing
NSAID: Nonsteroidal Anti-inflammatory Drug
OR: Odds Ratio
RF: Rheumatoid Factor
ROC: Receiver Operating Characteristic
SD: Sjögren’s Disease
